# The Maximal Vertical Pocket Better Identifies Pregnancies with Polyhydramnios at Increased Risk of Severe Neonatal Morbidity than the Amniotic Fluid Index: A Retrospective Cohort Study

**DOI:** 10.3390/jcm15176932

**Published:** 2026-09-07

**Authors:** Efrat Shekel, Gali Gordon, Nir Cohen, Dina Weinstein Mezan, Michal Lipschuetz, Doron Kabiri

**Affiliations:** 1 Department of Obstetrics and Gynecology, Hadassah University Medical Center, Ein Karem Campus, Jerusalem 9112001, Israel; 2 Henrietta Szold School of Nursing, Faculty of Medicine, Hebrew University, Jerusalem 9112102, Israel

**Keywords:** polyhydramnios, maximal vertical pocket, amniotic fluid index, neonatal morbidity

## Abstract

**Background:** Polyhydramnios is associated with adverse perinatal outcomes and is commonly assessed using either the amniotic fluid index (AFI) or the maximal vertical pocket (MVP). Although both methods are accepted in clinical practice, they frequently classify the same pregnancy differently, and their relative ability to predict adverse neonatal outcomes remains uncertain. **Methods:** We conducted a retrospective cohort study of pregnancies delivered at a tertiary university medical center between 2010 and 2025. Pregnancies with an AFI or MVP measurement within two weeks before delivery were eligible. Pregnancies complicated by diabetes mellitus or major fetal anomalies were excluded. Polyhydramnios severity was classified separately according to AFI and MVP criteria. The primary outcome was a composite of severe neonatal morbidity including neonatal intensive care unit admission, umbilical artery pH < 7.1, 5 min Apgar score < 7, or perinatal death. Separate multivariable logistic regression models were constructed for AFI- and MVP-based severity classifications. **Results:** Among 32,928 pregnancies, 3146 (9.6%) had polyhydramnios. Moderate-to-severe polyhydramnios defined by MVP was independently associated with severe neonatal morbidity (adjusted odds ratio [aOR] 2.47, 95% confidence interval [CI] 1.57–3.90; *p* < 0.001), whereas AFI-based severity classification was not (aOR 1.44, 95% CI 0.98–2.12; *p* = 0.07). Severe neonatal morbidity increased progressively with increasing MVP measurements. Mild polyhydramnios, regardless of the measurement method, was not associated with increased neonatal morbidity. Similar findings were observed in sensitivity analyses. **Conclusions:** MVP-based severity classification identified pregnancies at increased risk of severe neonatal morbidity, whereas AFI-based classification did not, at their conventional thresholds. These findings suggest that MVP may provide better risk stratification in pregnancies complicated by polyhydramnios and support prospective studies to validate its role in clinical management.

## 1. Introduction

Polyhydramnios, defined as an excessive accumulation of amniotic fluid, complicates approximately 1–2% of pregnancies and is associated with an increased risk of adverse maternal and neonatal outcomes. Although most cases are idiopathic, recognized causes include maternal diabetes mellitus, fetal structural or genetic anomalies, congenital infection, and fetal anemia. Identifying an underlying etiology is therefore a key component of the clinical evaluation [1].

Polyhydramnios has been associated with several adverse maternal and neonatal outcomes. Complications such as preterm birth, fetal macrosomia, malpresentation, cord prolapse, postpartum hemorrhage, neonatal intensive care unit (NICU) admission, and perinatal death have consistently been reported. The risk of adverse outcomes generally increases with disease severity [2,3,4,5,6,7,8,9,10]. While labor complications, such as preterm birth, umbilical cord prolapse, placental abruption and postpartum hemorrhage, can be explained by the effect of overdistention of the uterus, neonatal outcomes are not as easy to understand. A suggested explanation is some degree of placental insufficiency caused by intra-uterine increased pressure affecting the fetus in a multi-systematic manner, causing an increased rate of NICU admission, low Apgar score, and intra-uterine demise [11].

While recognized causes of polyhydramnios independently increase neonatal morbidity, idiopathic cases are much less understood. The effect of idiopathic polyhydramnios, which represents most cases, is particularly important for determining whether increased amniotic fluid volume itself identifies pregnancies at increased neonatal risk. Current evidence suggests that idiopathic polyhydramnios is associated with increased neonatal morbidity and mortality overall, although the magnitude of risk appears to vary considerably according to the severity of polyhydramnios and the neonatal outcome examined [7].

While the association of polyhydramnios with adverse neonatal outcomes becomes clearer with studies performed, the inconclusive guidelines regarding special monitoring, surveillance and the need for induction remain an issue. Furthermore, the severity of polyhydramnios has been suggested as a major factor affecting the risk of complications and adverse outcomes, but the technique used for assessment varies.

Amniotic fluid volume is routinely assessed using two accepted sonographic methods: the amniotic fluid index (AFI) and the maximal vertical pocket (MVP). AFI is calculated by summing the deepest vertical pocket in each of the four uterine quadrants, whereas MVP measures only the single deepest pocket. Polyhydramnios is conventionally defined as an AFI ≥ 250 mm or an MVP ≥ 80 mm and is further classified as mild, moderate, or severe using established thresholds [12,13].

Although both methods are considered acceptable in clinical practice, they frequently classify the same pregnancy differently. This discrepancy reflects the different measurement principles of the two methods. This discrepancy may influence disease severity classification, patient counseling, antenatal surveillance, and obstetric management.

In pregnancies complicated by oligohydramnios, randomized studies and systematic reviews have demonstrated that MVP reduces unnecessary obstetric intervention without compromising perinatal outcomes and is therefore the preferred method of assessment [14,15]. In contrast, the optimal method for evaluating polyhydramnios remains uncertain. Few studies have directly compared the prognostic performance of AFI and MVP; however, it remains unclear whether one method more accurately identifies pregnancies at increased risk of adverse neonatal outcomes [16,17].

Clarifying the prognostic value of these two methods could improve risk stratification and help tailor prenatal surveillance while avoiding unnecessary intervention in low-risk pregnancies. Therefore, we aimed to compare the ability of MVP- and AFI-based classification of polyhydramnios to identify pregnancies at increased risk of severe neonatal morbidity.

## 2. Materials and Methods

### 2.1. Study Design and Population

This retrospective cohort study was conducted using the electronic medical records of the two campuses of a tertiary university medical center. The study included pregnancies delivered between January 2010 and March 2025 in which amniotic fluid volume had been assessed by either the AFI or the MVP within two weeks before delivery, aiming to keep the amniotic fluid amount measurements as accurate as possible during the actual time of delivery. Ultrasound examinations were performed according to the departmental third-trimester ultrasound protocol by certified sonographers or obstetricians.

Polyhydramnios was defined as an AFI ≥ 250 mm and/or an MVP ≥ 80 mm. Pregnancies with normal amniotic fluid volume served as the reference group. Pregnancies complicated by pregestational or gestational diabetes mellitus, as well as major fetal anomalies (structural, genetic, or congenital infection-related), were excluded to evaluate idiopathic polyhydramnios, as much as possible, while minimizing the effect of other conditions which are potential confounders.

Polyhydramnios severity was classified according to established criteria as mild (AFI 250–299 mm or MVP 80–119 mm) or moderate-to-severe (AFI ≥ 300 mm or MVP ≥ 120 mm) [12].

The study was conducted in accordance with the Declaration of Helsinki and approved by the Institutional Review Board and Ethics Committee of Hadassah Medical Center (HMO-0079-23).

### 2.2. Outcomes

The primary outcome was a composite of severe neonatal morbidity, defined as the occurrence of at least one of the following: neonatal intensive care unit (NICU) admission, umbilical artery pH < 7.1, 5 min Apgar score < 7, or perinatal death.

Secondary outcomes included individual maternal and neonatal outcomes and a broader composite adverse maternal or neonatal outcome comprising preterm birth, birth weight < 2500 g, postpartum hemorrhage, and the components of the primary neonatal composite.

### 2.3. Statistical Analysis

Continuous variables were compared using the Mann–Whitney U test, and categorical variables were compared using the chi-square or Fisher’s exact test, as appropriate.

Separate multivariable logistic regression models were constructed for AFI- and MVP-based severity classifications to analyze the association between polyhydramnios severity and the primary outcome. The multivariable logistic regression model was adjusted for maternal age, parity, hypertensive disorders of pregnancy, and assisted reproductive technology.

Trends in the primary outcome across the full AFI and MVP ranges were evaluated using Spearman’s rank correlation. Wilson 95% confidence intervals (CI) were calculated for proportions.

A prespecified sensitivity analysis repeated the primary analysis after including pregnancies complicated by diabetes mellitus or major fetal anomalies. Comparisons of polyhydramnios severity for the primary outcome were prespecified. Analyses of secondary outcomes were considered exploratory, and *p* values were not adjusted for multiple comparisons. All statistical tests were two-sided, and *p* < 0.05 was considered statistically significant.

### 2.4. Comparison Between the Two Measurement Methods

Because each scale was applied with its own conventional severity threshold, and because those thresholds classify different proportions of the cohort, three additional analyses were performed to compare the methods directly rather than by inference from two separate models.

First, among pregnancies in which both measurements were recorded, the moderate-to-severe categories defined by each scale were entered simultaneously into a single logistic model, so that each was estimated conditional on the other.

Second, the difference between the two method-specific odds ratios was tested formally, using a method-by-severity interaction term and a bootstrap of the ratio of the two odds ratios (4000 resamples), which accounts for the same women contributing to both estimates.

Third, because the conventional thresholds are not equivalent in the proportion of women they classify, each scale was re-cut at the value that flagged the same proportion of its own polyhydramnios group as the other scale’s conventional threshold, and the analysis was repeated at these prevalence-matched cut-offs.

## 3. Results

### 3.1. Study Population

**Figure 1:**
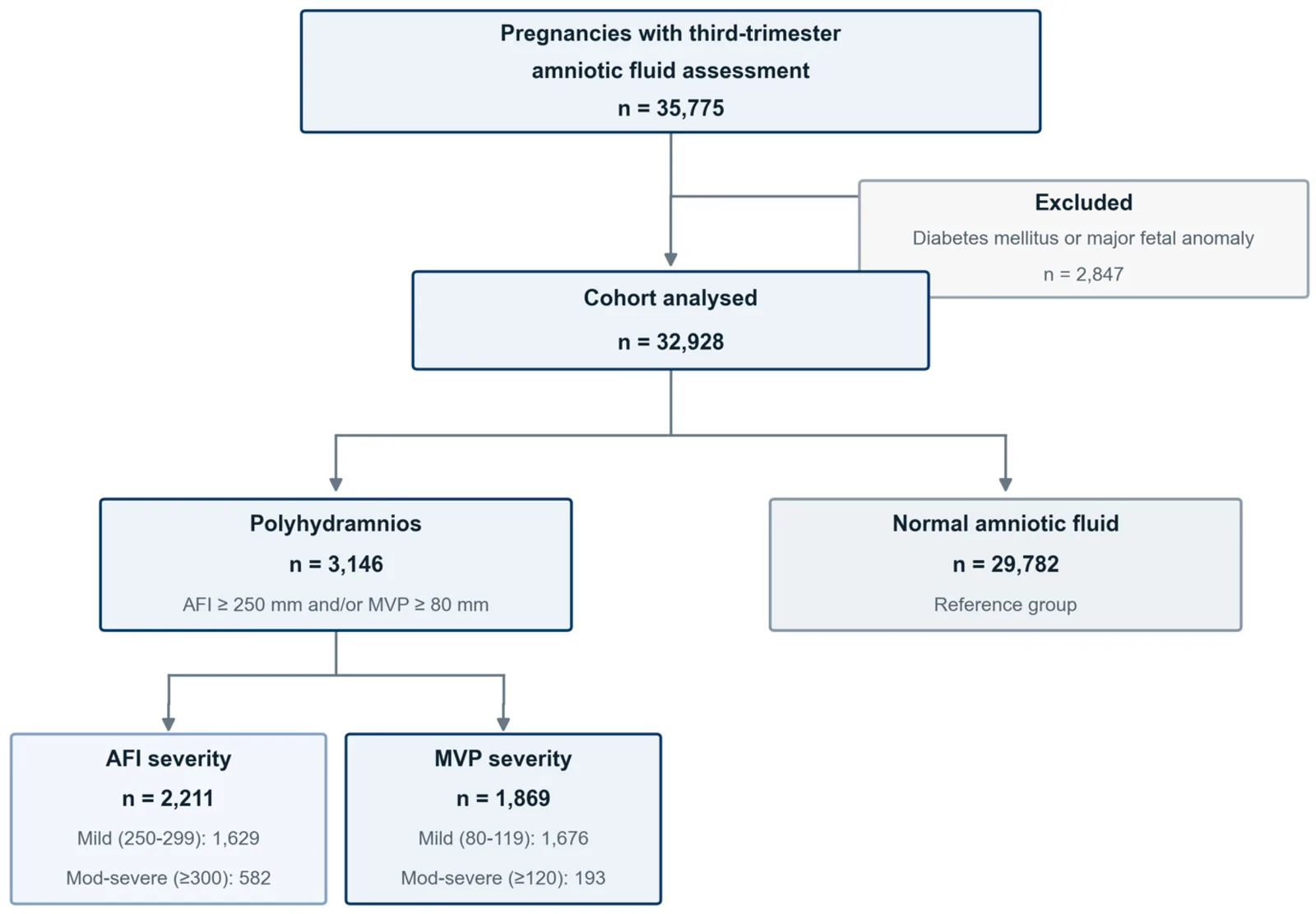
Study design and group division. Assembly of the study cohort. The AFI and MVP groups overlap; 934 pregnancies met both definitions.

A total of 35,775 pregnancies with an amniotic fluid measurement within two weeks before delivery were identified. After excluding 2847 pregnancies complicated by diabetes mellitus or major fetal anomalies, 32,928 pregnancies were included in the final analysis. Of these, 3146 (9.6%) had polyhydramnios and 29,782 (90.4%) had normal amniotic fluid volume (Figure 1).

Baseline maternal characteristics are presented in Table 1. Compared with women with normal amniotic fluid volume, women with polyhydramnios were slightly older (30.5 vs. 29.9 years) and more frequently multiparous. The rate of assisted reproductive technology was also slightly higher in the polyhydramnios group, whereas the hypertensive disorder rate was lower. The rates of previous cesarean delivery and smoking were similar between both groups.

Pregnancies complicated by polyhydramnios were more likely to undergo labor induction (36.7% vs. 28.0%) and cesarean delivery (25.4% vs. 21.6%). Preterm birth (6.1% vs. 7.1%) and placental abruption (0.5% vs. 1.1%) were slightly less frequent in pregnancies with polyhydramnios. Pregnancies complicated by polyhydramnios also had higher birth weights (median 3510 vs. 3238 g), a higher rate of macrosomia (14.2% vs. 6.0%) and fewer neonates weighing < 2500 g (6.5% vs. 8.9%). The rates of obstetric anal sphincter injury (OASI) and umbilical cord prolapse were similar between both groups. Pregnancy and neonatal outcomes are shown in Table 2.

### 3.2. Primary Outcome

The incidence of severe neonatal morbidity was 5.6% among pregnancies with normal amniotic fluid volume.

When polyhydramnios severity was classified using MVP, the primary outcome occurred in 6.3% of pregnancies with mild polyhydramnios and 14.0% of those with moderate-to-severe polyhydramnios (27/193), corresponding to an odds ratio of 2.41 (95% CI 1.53–3.78; *p* < 0.001) (Table 3).

The multivariable logistic regression model (adjusted for maternal age, parity, hypertensive disorders and assisted reproductive technology) corresponded to an adjusted odds ratio (aOR) of 2.47 (95% CI 1.57–3.90; *p* < 0.001).

In contrast, AFI-based severity classification was not associated with severe neonatal morbidity. The primary outcome occurred in 5.3% of pregnancies with mild polyhydramnios and 7.0% of those with moderate-to-severe polyhydramnios, corresponding to a non-significant odds ratio of 1.36 (95% CI 0.93–2.00; *p* = 0.120) (Table 4).

After adjustment for maternal age, parity, hypertensive disorders of pregnancy, and assisted reproductive technology, the aOR was 1.44 (95% CI 0.98–2.12; *p* = 0.07).

### 3.3. Trend Analysis

**Figure 2:**
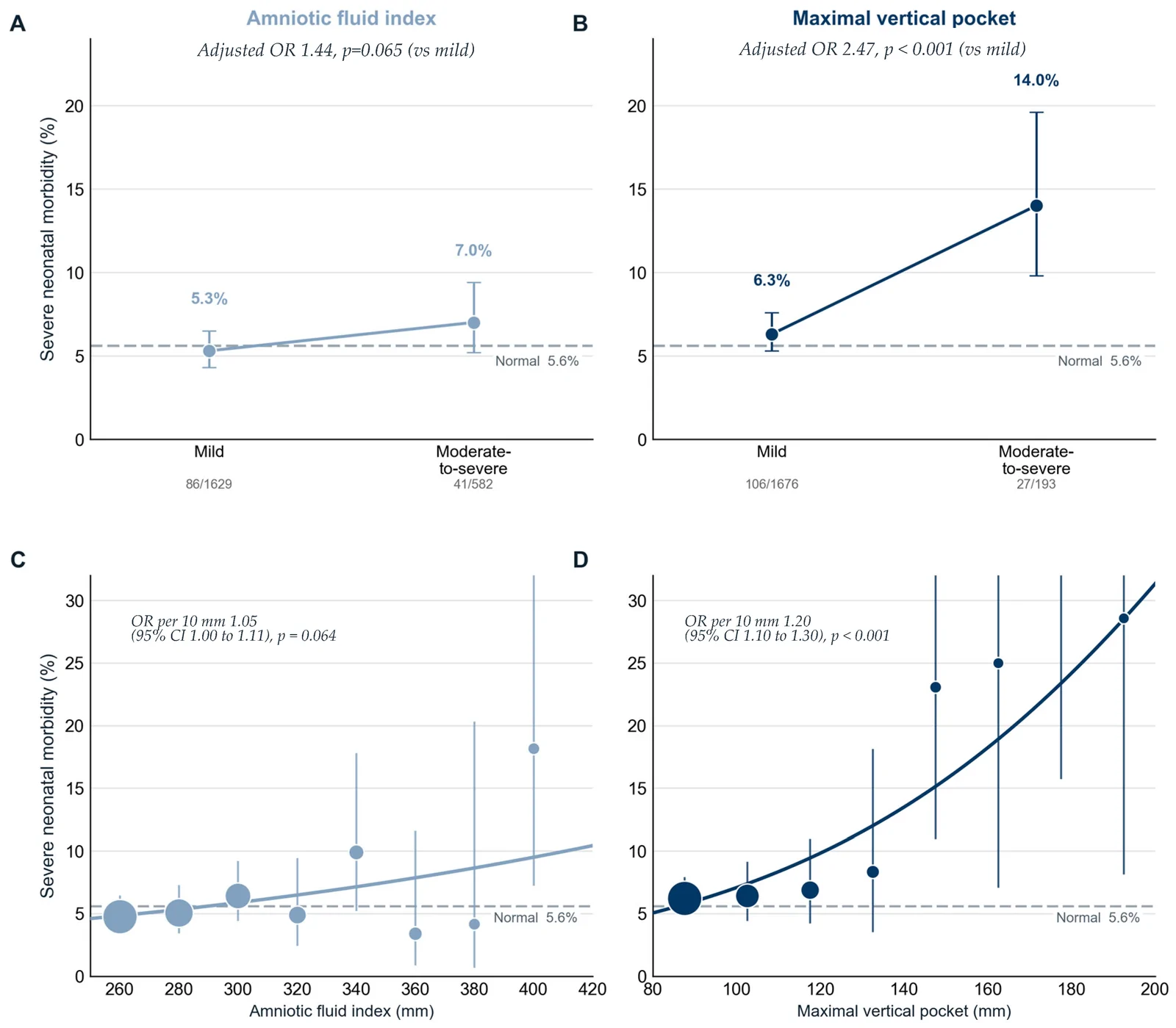
Trend analysis for severe neonatal morbidity according to method and severity of polyhydramnios. Panels (**A**) (AFI) and (**B**) (MVP) show the rate by severity category (mild versus moderate-to-severe) for each scale, with the adjusted odds ratio relative to mild disease. Panels (**C**,**D**) show the continuous relationship between the measured value and severe neonatal morbidity, with the points as the observed rate per bin, and the size of each point is proportional to the number of observations (Wilson 95% CI). The dashed line indicates the rate in pregnancies with a normal amniotic fluid volume.

The incidence of severe neonatal morbidity increased progressively with increasing MVP measurements, reaching 28.2% among pregnancies with severe polyhydramnios (MVP of at least 160 mm). In contrast, no significant trend was observed across AFI categories (Figure 2).

**Figure 3:**
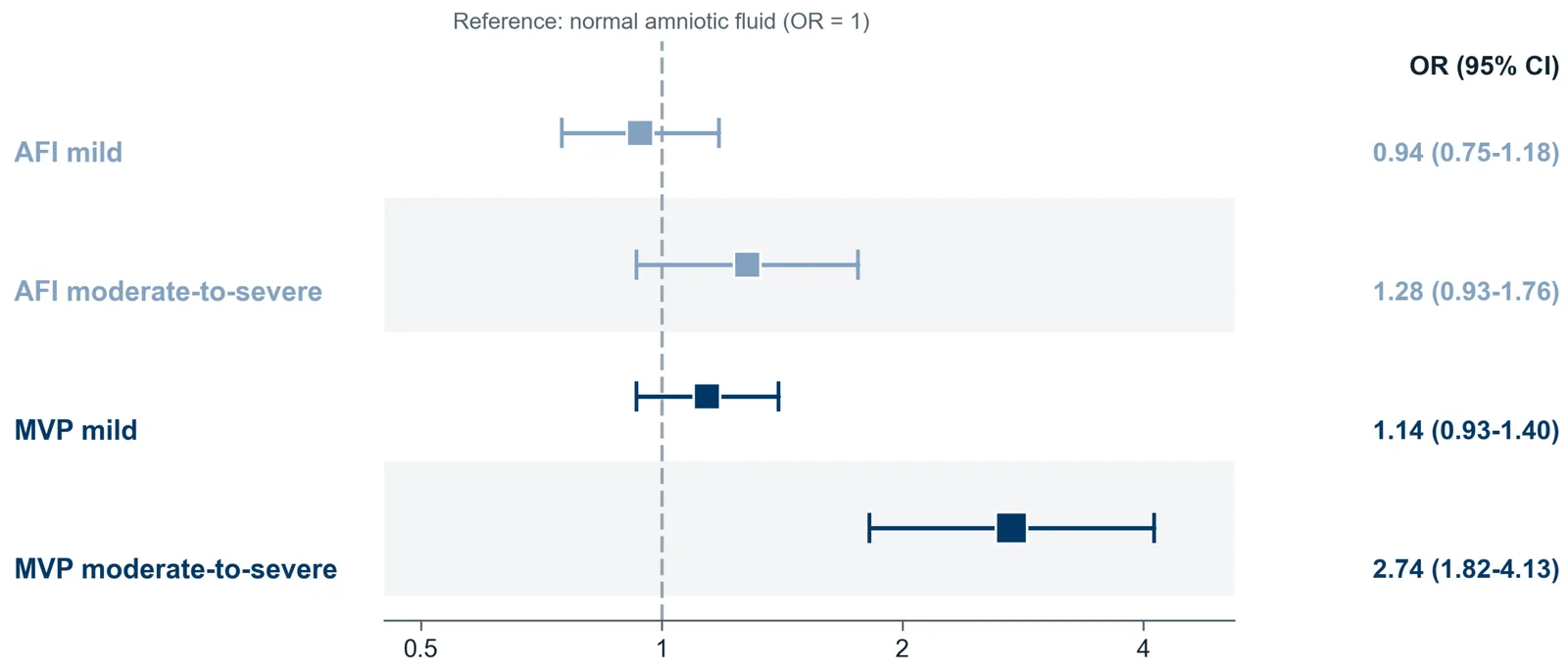
Odds ratio for severe neonatal morbidity according to method and severity of polyhydramnios versus normal amniotic fluid. Odds ratio of severe neonatal morbidity for each polyhydramnios severity category versus a normal amniotic fluid volume; only moderate-to-severe polyhydramnios defined by the MVP is significantly increased.

Overall, only moderate-to-severe polyhydramnios classified by MVP was associated with an increased risk of severe neonatal morbidity. Mild polyhydramnios, regardless of the measurement method, and all AFI severity categories showed rates comparable to those of pregnancies with normal amniotic fluid volume (Figure 3).

### 3.4. Secondary Outcomes

Secondary outcome analyses, considering broader composite adverse maternal and neonatal outcomes, showed findings consistent with those of the primary outcome.

When considering preterm birth, low birth weight (<2500 g) and postpartum hemorrhage, in addition to neonatal outcomes mentioned before, moderate-to-severe polyhydramnios defined by MVP was associated with higher rates of adverse maternal and neonatal outcomes (31.1% vs. 22.4%), compared with mild polyhydramnios (odds ratio of 1.56 (95% CI 1.13–2.16; *p* = 0.009)) (Table 5).

In contrast, AFI-based severity classification was not associated with most secondary outcomes, nor did it show a significant increase with higher polyhydramnios severity (odds ratio of 1.10 (95% CI 0.87–1.39; *p* = 0.430)).

### 3.5. Sensitivity Analysis

Including pregnancies complicated by diabetes mellitus or major fetal anomalies did not materially change the results. Moderate-to-severe polyhydramnios defined by MVP remained independently associated with severe neonatal morbidity, with effect estimates similar to those observed in the primary analysis.

### 3.6. Direct Comparison of the Two Methods

The conventional thresholds classified different proportions of the cohort. An MVP of at least 120 mm identified 193 of 1869 pregnancies (10.3%), with a severe neonatal composite rate of 14.0%, whereas an AFI of at least 300 mm identified 582 of 2211 pregnancies (26.3%), with a rate of 7.0%.

Among the 1543 pregnancies with both measurements (84 events), entering the two moderate-to-severe categories simultaneously gave an odds ratio of 2.17 (1.09–4.35; *p* = 0.028) for the MVP and 1.48 (0.87–2.54; *p* = 0.149) for the AFI. The formal comparison between the methods, however, did not reach significance: the ratio of the two odds ratios was 1.45 (95% CI 0.75–2.66; *p* = 0.235) and the method-by-severity interaction was not significant (*p* = 0.11).

At prevalence-matched cut-offs, the two scales performed similarly. An AFI of at least 326 mm, which flagged 10.6% of its polyhydramnios group and therefore matched the proportion flagged by an MVP of at least 120 mm, was associated with a rate of 11.1% and an odds ratio of 2.31 (1.47–3.64). Conversely, an MVP of at least 102 mm, matched to the 26.3% flagged by an AFI of at least 300 mm, was associated with a rate of 9.7% and an odds ratio of 1.66 (1.15–2.39).

## 4. Discussion

### 4.1. Principal Findings

The principal finding of this study is that the association between polyhydramnios severity and severe neonatal morbidity depends on the sonographic method used for assessment. Moderate-to-severe polyhydramnios defined by MVP, but not AFI, was independently associated with severe neonatal morbidity. The association remained significant after adjustment for potential confounders and persisted in the sensitivity analysis that included pregnancies complicated by diabetes mellitus or major fetal anomalies. Furthermore, the incidence of severe neonatal morbidity increased progressively with increasing MVP measurements.

In contrast, AFI-based severity classification was not associated with severe neonatal morbidity. In addition, mild polyhydramnios defined by either method was not associated with a higher rate of neonatal morbidity, compared with pregnancies with normal amniotic fluid volume.

Having said that, although the conventional use of MVP showed better correlation to severe neonatal outcomes, the direct comparison between the two methods was not itself statistically significant, and at prevalence-matched cut-offs, the two scales behaved similarly. These analyses refine the interpretation of the principal finding.

The finding is therefore best understood as a property of where each conventional threshold sits on its own distribution, rather than as evidence that one sonographic technique measures risk better than the other.

Accordingly, in our view, the data supports a narrower, yet important clinical conclusion. The conventional MVP threshold isolates a small, markedly higher-risk subgroup, whereas the conventional AFI threshold identifies a group two and a half times larger, in which the excess risk is correspondingly diluted.

### 4.2. Comparison with Previous Studies

Polyhydramnios is a well-established risk factor for adverse perinatal outcomes, particularly in more severe disease [7,8,9,10,18]. However, few studies have directly compared the prognostic performance of AFI and MVP in pregnancies with polyhydramnios. Previous studies have shown that AFI and MVP identify overlapping but non-identical populations because AFI classifies more pregnancies as polyhydramnios than MVP [16,17]. In contrast, the evidence is stronger for oligohydramnios, where randomized studies and systematic reviews support the use of MVP because it reduces unnecessary obstetric intervention without compromising perinatal outcomes [14,15].

Our findings extend the existing literature by demonstrating that MVP-based severity classification better discriminates pregnancies at increased risk of severe neonatal morbidity than AFI-based classification. The observed trend further supports the prognostic value of MVP in this setting.

### 4.3. Clinical Implications

The mechanisms underlying the differing prognostic performance of AFI and MVP remain uncertain. Because AFI combines measurements from four uterine quadrants, it may dilute localized excess amniotic fluid, whereas MVP reflects the largest fluid pocket and may better capture clinically relevant uterine overdistension. The result of this gap is exemplified by the different thresholds for the same underlying condition as presented. Although this hypothesis requires further investigation, this observation carries a practical implication that extends beyond the choice of scale.

Because of the different thresholds, the two methods necessarily label different numbers of pregnancies as high-risk, along with the implications of it. A more inclusive threshold can ensure better identification of ultimately complicated pregnancies, with the cost of labeling more pregnancies as high-risk, most of which will have an uncomplicated outcome.

As for many other findings and diagnoses, the question is not only which measurement is correct, but which operating point best balances detection against over-labeling in each clinical setting.

From a clinical perspective, moderate-to-severe polyhydramnios defined by MVP identified pregnancies at increased risk of severe neonatal morbidity, whereas AFI-based severity classification did not. These findings suggest that MVP may be more useful for identifying pregnancies that require closer antenatal surveillance.

### 4.4. Strengths and Limitations

The strengths of this study include its large cohort, comprising more than 32,000 pregnancies and over 3000 cases of polyhydramnios, the use of standardized severity definitions, and a clinically meaningful primary outcome. The findings remained consistent across prespecified sensitivity analyses. Although the direct comparison between the two measurement methods did not reach statistical significance, the overall findings consistently suggested that the MVP method identified a subgroup at increased risk for severe neonatal morbidity, whereas the AFI method did not.

Several limitations should be acknowledged. First, the retrospective design is inherently susceptible to residual confounding despite multivariable adjustment. Second, the number of pregnancies with moderate-to-severe polyhydramnios, particularly those with very high maximal vertical pocket measurements, was relatively small, limiting the precision of estimates for these subgroups. Third, NICU admission was the principal contributor to the composite outcome of severe neonatal morbidity. Because admission thresholds and local clinical practices vary across institutions, this component may have influenced the observed magnitude of the association. Finally, the head-to-head comparison was limited to the 1543 pregnancies (49% of the polyhydramnios cohort) in which both amniotic fluid measurements were available. Furthermore, the prevalence-matched severity thresholds were derived within this cohort and therefore require external validation before they can be adopted in clinical practice.

## 5. Conclusions

Among pregnancies with polyhydramnios, MVP-based severity classification showed better association with severe neonatal morbidity, in comparison with AFI-based classification.

Mild polyhydramnios, regardless of the measurement method, was not associated with an increased risk compared with normal amniotic fluid volume. These findings suggest that MVP-based severity classification may improve identification of pregnancies at increased neonatal risk and support further prospective studies to validate its role in clinical risk stratification.

## Figures and Tables

**Table 1:** Demographic and clinical characteristics.

Characteristic	Polyhydramnios (n = 3146)	Normal Amniotic Fluid (n = 29,782)	*p* Value
Maternal age, years	30.5 [26–35]	29.9 [25–34]	*p* < 0.001
Gravidity	3.8 ± 2.7	3.4 ± 2.5	*p* < 0.001
Parity	3.0 ± 2.2	2.6 ± 2.1	*p* < 0.001
Nulliparous	24.8%	30.5%	*p* < 0.001
Previous cesarean delivery	16.9%	17.2%	*p* = 0.747
Assisted reproductive technology	6.5%	5.3%	*p* = 0.005
Smoking	4.7%	4.1%	*p* = 0.140
Hypertensive disorder	2.3%	3.0%	*p* = 0.017

Data are median [interquartile range], mean ± standard deviation, or percentage of pregnancies with the variable recorded.

**Table 2:** Pregnancy and neonatal outcomes.

Outcome	All Polyhydramnios (n = 3146)	Normal Amniotic Fluid (n = 29,782)	*p* Value
Induction of labor	36.7%	28.0%	*p* < 0.001
**Mode of delivery** - **Vaginal delivery** - **Operative delivery** - **Cesarean delivery**	66.6% 7.9% 25.4%	71.0% 7.4% 21.6%	*p* < 0.001 *p* = 0.268 *p* < 0.001
Preterm birth < 37 weeks	6.1%	7.1%	*p* = 0.043
Placental abruption	0.5%	1.1%	*p* = 0.015
Postpartum hemorrhage	4.2%	4.1%	*p* = 0.887
Birth weight, grams	3510 [3132–3814]	3238 [2880–3576]	*p* < 0.001
Macrosomia ≥ 4000 g	14.2%	6.0%	*p* < 0.001
Low birth weight < 2500 g	6.5%	8.9%	*p* < 0.001
OASI	0.3%	0.6%	*p* = 0.058
Umbilical cord prolapse	0.2%	0.1%	*p* = 0.104

Data are median [interquartile range] or percentage of pregnancies with the variable recorded. For mode of delivery, the *p* value refers to each mode of delivery individually.

**Table 3:** Neonatal outcomes by severity of polyhydramnios using the MVP method.

Outcome	Normal Amniotic Fluid (n = 29,782)	Mild (n = 1676)	Moderate-to-Severe (n = 193)	Odds Ratio (95% CI) Moderate-to-Severe vs. Mild
NICU admission	4.2%	4.4%	13.0%	3.22 (1.99–5.21), *p* < 0.001
Cord arterial pH < 7.1	2.4%	3.8%	3.9%	1.04 (0.31–3.51), *p* = 1.000
5 min Apgar < 7	0.4%	0.5%	0.0%	not estimable
Perinatal death	0.0%	0.1%	0.0%	not estimable
Severe neonatal composite outcome	5.6%	6.3%	14.0%	2.41 (1.53–3.78), *p* < 0.001

The severe neonatal composite outcome comprised NICU admission, umbilical cord arterial pH below 7.1, a five-minute Apgar score below 7, or perinatal death. The odds ratio compares moderate-to-severe with mild polyhydramnios. NICU, neonatal intensive care unit.

**Table 4:** Neonatal outcomes by severity of polyhydramnios using the AFI method.

Outcome	Normal Amniotic Fluid (n = 29,782)	Mild (n = 1629)	Moderate-to-Severe (n = 582)	Odds Ratio (95% CI) Moderate-to-Severe vs. Mild
NICU admission	4.2%	3.2%	5.2%	1.65 (1.04–2.61), *p* = 0.040
Cord arterial pH < 7.1	2.4%	3.6%	2.8%	0.78 (0.33–1.81), *p* = 0.688
5 min Apgar < 7	0.4%	0.9%	0.9%	1.00 (0.36–2.79), *p* = 1.000
Perinatal death	0.0%	0.1%	0.0%	not estimable
Severe neonatal composite outcome	5.6%	5.3%	7.0%	1.36 (0.93–2.00), *p* = 0.120

The severe neonatal composite outcome comprised NICU admission, umbilical cord arterial pH below 7.1, a five-minute Apgar score below 7, or perinatal death. The odds ratio compares moderate-to-severe with mild polyhydramnios. NICU, neonatal intensive care unit.

**Table 5:** Broad composite adverse maternal and neonatal outcomes by severity of polyhydramnios based on measuring method.

Measurement Method	Mild	Moderate-to-Severe	Odds Ratio (95% CI) Moderate-to-Severe vs. Mild
AFI	19.3%	20.8%	1.10 (0.87–1.39), *p* = 0.430
MVP	22.4%	31.1%	1.56 (1.13–2.16), *p* = 0.009

The composite adverse maternal or neonatal outcomes comprised preterm birth, low birth weight below 2500 g, a five-minute Apgar score below 7, umbilical cord arterial pH below 7.1, admission to a neonatal intensive care unit, postpartum hemorrhage, or perinatal death. The odds ratio compares moderate-to-severe with mild polyhydramnios on the same scale.

## Data Availability

The original contributions presented in this study are included in the article. Further inquiries can be directed to the corresponding author.

## Abbreviations

The following abbreviations are used in this manuscript:

AFI	Amniotic Fluid Index
MVP	Maximal Vertical Pocket
NICU	Neonatal Intensive Care Unit
SD	Standard Deviation
CI	Confidence Interval
aOR	Adjusted Odds Ratio
